# Supplementary material for: Prevalence of Ranavirus Infection in Three Anuran Species across South Korea
Source: Viruses. 2022 May 17;14(5):1073. doi: 10.3390/v14051073 (PMC9148164; doi:10.3390/v14051073)

Supplementary Materials Figure S1: The representative qPCR results of *D. japonicus* (A,B) collected at Haenam, *P. nigromaculatus* (C,D) collected at Pohang, and *L. catesbeianus* (E,F) collected at Gimchoen, and the photographs of representative gel electrophoresis of the MCP fragment (~520 bp), which confirmed the qPCR result again by sequencing the fragment (G,H) in the further study.

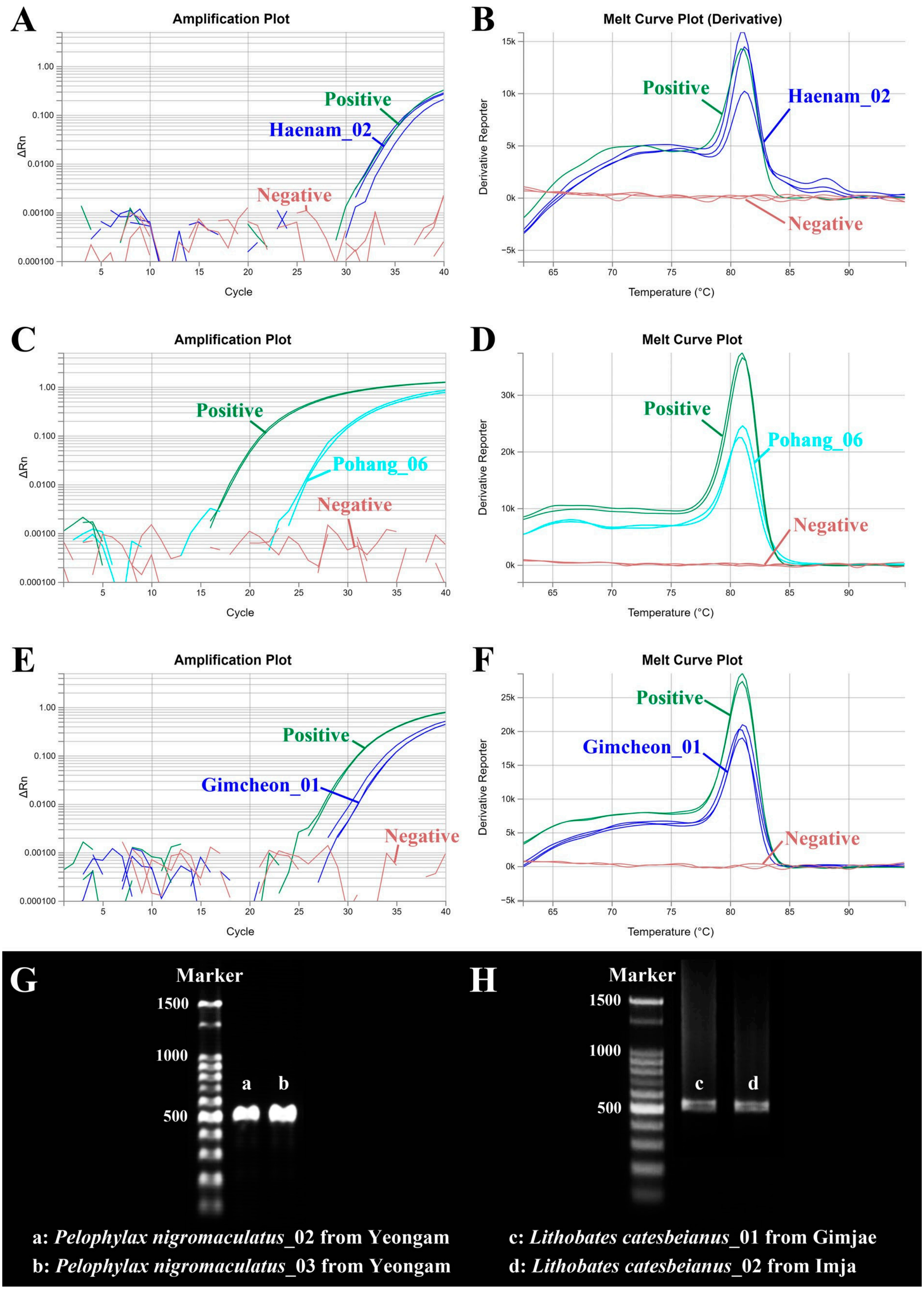

Supplement: Supplementary file 1 [file viruses-14-01073-s001.zip › viruses-1720076-supplementary.pdf]
